# Real-World Clinical Outcomes and Treatment Patterns Among Black and Non-Black Patients With Prostate Cancer Initiated on Apalutamide in a Urology Setting

**DOI:** 10.36469/001c.121233

**Published:** 2024-08-19

**Authors:** Benjamin H. Lowentritt, Carmine Rossi, Erik Muser, Frederic Kinkead, Bronwyn Moore, Patrick Lefebvre, Dominic Pilon, Shawn Du

**Affiliations:** 1 Chesapeake Urology Associates; 2 Analysis Group, Inc., Montréal, Canada; 3 Janssen Scientific Affairs, LLC, Horsham, Pennsylvania, USA

**Keywords:** metastatic hormone-sensitive prostate cancer, non-metastatic hormone-relapsed prostate cancer, race, mCSPC, mHSPC, nmCRPC, nmHRPC, metastatic castration-sensitiveprostate cancer, non-metastatic castration-resistant prostate cancer

## Abstract

**Background:** The use of androgen receptor signaling inhibitors, including apalutamide, in combination with androgen deprivation therapy is recommended for the treatment of metastatic castration-sensitive prostate cancer (mCSPC) and non-metastatic castration-resistant prostate cancer (nmCRPC).

**Objective:** To describe real-world treatment patterns and clinical outcomes among patients with mCSPC or nmCRPC who initiated apalutamide in the United States.

**Methods:** A retrospective cohort study of patients with mCSPC or nmCRPC who initiated apalutamide was conducted using electronic medical record data from US community-based urology practices (Feb. 1, 2017–April 1, 2022). Persistence with apalutamide was reported at 6-, 12-, and 18-months post treatment initiation. Clinical outcomes described up to 24 months after apalutamide initiation using Kaplan-Meier analyses included progression to castration resistance, castration resistance-free survival (CRFS), and metastasis-free survival (MFS). Outcomes were reported separately based on mCSPC or nmCRPC status and race (ie, Black or non-Black).

**Results:** This study included 589 patients with mCSPC (mean age, 75.9 years) and 406 patients with nmCRPC (mean age, 78.8 years). Using a treatment gap of >90 days, persistence with apalutamide at 12 months remained high for both the mCSPC (94.9%) and nmCRPC (92.7%) cohorts, and results were descriptively similar among Black and non-Black patients, and when a treatment gap of >60 days was considered. In patients with mCSPC, overall progression to castration resistance rates at 12 and 24 months were 20.9% and 33.5%, and overall CRFS rates were 76.2% and 62.0%, respectively. In patients with nmCRPC, overall MFS rates at 12 and 24 months were 89.7% and 75.4%, respectively. Rates of these clinical outcomes were descriptively similar between Black and non-Black patients.

**Discussion:** While clinical trials have demonstrated the efficacy and safety of apalutamide, there is limited real-world data describing treatment persistence and clinical outcomes among patients with mCSPC and nmCRPC who initiated apalutamide.

**Conclusions:** In this real-world study of patients with mCSPC or nmCRPC initiated on apalutamide, treatment persistence was high and apalutamide demonstrated robust real-world effectiveness with respect to progression to castration resistance, CRFS, and MFS, overall and among Black and non-Black patients.

## BACKGROUND

Prostate cancer (PC) is the most common type of cancer and second leading cause of cancer-related death among men in the United States, with an estimated 288 300 new cases and 34 700 deaths in 2023.^1,2^ Metastasis to the bone and other sites, including lymph nodes, lungs and the liver, contributes to PC-related mortality,^3,4^ with an estimated 5-year survival of 34.1% after metastasis development.^1^

Racial disparities are well-documented in patients with PC, including lower screening and higher incidence and mortality observed among Black men.^1,5^ Moreover, the American Urological Association/American Society for Radiation Oncology/Society of Urologic Oncology Guidelines for the treatment of metastatic castration-sensitive PC (mCSPC) and non-metastatic castration-resistant PC (nmCRPC) are informed by evidence from clinical trials,^6,7^ but Black men are underrepresented in clinical trials of PC therapies.^8^ Whether advanced PC therapies are equally effective among Black men remains an important knowledge gap which may have implications for treatment guidelines.

Apalutamide is an androgen receptor signaling inhibitor that, in combination with androgen deprivation therapy (ADT), was approved for the treatment of nmCRPC in February 2018 and mCSPC in September 2019, based on the results of the SPARTAN and TITAN phase 3 clinical trials, respectively.^9,10^ Results from the SPARTAN trial showed that apalutamide in combination with ADT was associated with a 72% reduction in metastasis progression or death, compared to ADT alone, in patients with nmCRPC.^11^ Similarly, results from the TITAN trial demonstrated that use of apalutamide and ADT was associated with a 52% reduction in radiographic progression or death, relative to ADT alone, in patients with mCSPC.^12^ Further analyses from the SPARTAN and TITAN trials have shown that patients receiving apalutamide plus ADT experienced improvements in patient-reported outcomes and maintenance of health-related quality of life compared to placebo plus ADT.^13,14^ In both the SPARTAN and TITAN trials, Black men comprised a small proportion of the overall sample, where 5.6% of SPARTAN patients and 1.8% of TITAN patients were Black.^13,14^

While apalutamide has demonstrated efficacy in the clinical trial setting, patients included in randomized controlled trials are highly selective and may not be representative of the general population of patients with advanced PC. Therefore, there is a need to characterize patients receiving apalutamide in real-world clinical practices and assess treatment effectiveness among a more heterogenous patient population. There are currently limited real-world data describing treatment patterns and clinical outcomes among patients with mCSPC or nmCRPC who initiated apalutamide in the US, or elsewhere, including race-specific information.^15,16^ Race-specific data are integral to reducing health disparities in clinical care settings by helping identify areas where quality of care can be improved for specific populations and address existing inequalities.^17^ Consequently, the aim of this study was to describe real-world treatment patterns and clinical outcomes evaluated up to two years after apalutamide initiation, including progression to castration resistance (CR), CR-free survival (CRFS), and metastasis-free survival (MFS), among patients with mCSPC or nmCRPC in the US, overall and stratified by race (ie, Black and non-Black).

## METHODS

### Data Source

Electronic medical record (EMR) data from Precision Point Specialty (PPS) Analytics were obtained as part of routine clinical care from community-based urology practices distributed across the United States (Feb. 1, 2017–April 1, 2022). Information on patient demographics, clinical characteristics (including metastasis dates, CR indicators) and laboratory tests (including longitudinal prostate-specific antigen [PSA] measurements) were available. In-office medication dispensing information was also available to a large subset of patients and provided information on apalutamide prescriptions (eg, fill date, quantity dispensed, days’ supply), which are not regularly available from EMR data sources, to allow an assessment of treatment patterns. Furthermore, the PPS database also abstracts information from clinical notes to capture information on dates and locations of metastasis and progression to CR, which may not be available in structured EMR data tables. Institutional review board approval was not necessary for this study, as data were de-identified and comply with the requirements of the Health Insurance Portability and Accountability Act.

### Study Design

A retrospective, longitudinal cohort study design was used. The first dispensation of apalutamide was defined as the index date. The 12-month period prior to the index date was defined as the baseline period. The time from the index date to the earliest of end of clinical activity (defined as the last record in any of the component EMR tables, eg, prescriptions, diagnoses, procedures, test results), end of data availability (April 1, 2022), or death was defined as the observation period. Patients were classified into mCSPC or nmCRPC cohorts, based on the presence or absence of metastasis and CR (described below).

### Patient Selection Criteria

Eligible participants included men at least 18 years of age with mCSPC or nmCRPC who had at least 1 in-office medication dispensation for apalutamide and demonstrated at least 12 months of clinical activity preceding the index date. To avoid potential survival bias, no minimum follow-up duration was required. Patients included in the mCSPC cohort were required to have evidence of metastatic disease, without CR, before or on the index date. Identification of metastasis was based on a PPS-provided indicator variable for the presence of bone, nodal, or visceral metastasis. Patients included in the nmCRPC cohort were required to have evidence of CR, without documented metastasis, before or on the index date.

CR was identified through a previously published algorithm incorporating the presence of ADT and rising PSA levels,^18^ or a PPS-provided indicator variable for CR. From the published algorithm,^18^ evidence of CR was based on 3 criteria: identification of hormone resistance on or before the index date, based on the presence of one or more *International Classification of Diseases, Tenth Revision, Clinical Modification* (ICD-10-CM) codes (ie, Z19.2); rising PSA levels post-surgical castration; and rising PSA levels post-medical castration. Further details regarding these criteria have previously been described.^16^ The PPS-provided indicator variable for CR was based on the following criteria: 2 consecutive rises in PSA while on continuous ADT therapy for more than 6 months, the presence of the ICD-10-CM diagnostic code for hormone resistance (Z19.2), or mention of CR from manual data entry by a clinical user or parsable encounter notes.

Participants were excluded if they had previously used another androgen receptor signaling inhibitor or radiopharmaceutical prior to or on the index date, or began apalutamide treatment prior to the US Food and Drug Administration approval dates for mCSPC (ie, 17 September 2019)^9^ or nmCRPC (ie, 14 February 2018).^10^ Concurrent use of ADT was not a requirement for inclusion in either the mCSPC or nmCRPC cohort.

### Study Measures and Outcomes

Patient demographic and clinical characteristics were assessed during the 12-month baseline period. Consistent with prior PC studies, treatment persistence during the observation period was defined as the percentage of patients without an apalutamide treatment gap of more than 60 days or more than 90 days by 6-, 12-, and 18-months post-index.^15,19^ Gaps in apalutamide treatment were measured from the end of the days of supply of the previous dispensation to the earliest of the next dispensation or the end of data availability. Treatment adherence during the observation period was measured by the proportion of days covered, which was defined as the sum of non-overlapping days of supply of apalutamide divided by the total length of time within an interval of 6, 12 and 18-months post-index. A patient was considered adherent to apalutamide if the proportion of days covered was at least 0.80. The following clinical outcomes were assessed during the observation period: (1) progression to CR, defined as the proportion of patients developing CR, up to 24 months post-index (only assessed among patients with mCSPC); (2) CRFS, defined as the proportion of patients without CR or death, up to 24 months post-index (only assessed among patients with mCSPC); and (3) MFS, defined as the proportion of patients without metastasis progression (ie, confirmed bone, nodal, or visceral metastasis) or death, up to 24 months post-index (only assessed among patients with nmCRPC).

### Statistical Analysis

All analyses were descriptive and reported separately by study cohort (ie, mCSPC or nmCRPC) and race (ie, Black or non-Black [ie, White or Other, details on the composition of the Other race category were not available in the data source]). Patients with an unknown race were included in the nonstratified analyses but were excluded from the race-stratified analyses. Complete observation during each specific time period was a requirement for inclusion of patients in each persistence and adherence calculation (ie, at 6, 12, and 18 months). Rates of progression to CR, CRFS, and MFS were described using Kaplan-Meier analysis, and patients without an event were censored at the earliest of end of clinical activity or end of data availability (for all outcomes), or death (for progression to CR). As this analysis was descriptive, no adjustments for differences in baseline characteristics between Black and non-Black patients were made.

## RESULTS

### Baseline Characteristics

Data from 589 patients with mCSPC and 406 patients with nmCRPC treated with apalutamide were identified (**Supplementary Figure S1**). Baseline characteristics are summarized in **Table 1**. Among patients with mCSPC, 98 (16.6%) were Black and 424 (72.0%) were non-Black, while in the nmCRPC cohort, 74 (18.2%) were Black and 301 (74.1%) were non-Black. The mean age was 75.9 years (Black, 72.4 years; non-Black, 76.8 years) in patients with mCSPC and 78.8 years (Black, 76.2 years; non-Black, 79.3 years) in patients with nmCRPC. For patients with mCSPC, the median time between metastasis and apalutamide initiation was 1.5 months overall, with a numerically longer median time observed in Black patients than in non-Black patients (2.2 months vs 1.5 months). For patients with nmCRPC, the median time between CR and apalutamide initiation was 8.7 months overall, with a median time that was also numerically longer in Black patients than in non-Black patients (15.6 months vs 7.8 months).

**Table 1.** Baseline Characteristics

	**mCSPC**			**nmCRPC**		
	**All Patients, N = 589**	**Black Patients, N = 98**	**Non-Black Patients, N = 424**	**All Patients, N = 406**	**Black Patients, N = 74**	**Non-Black Patients, N = 301**
Age, mean ± SD (median)	75.9 ± 7.7 (76.0)	72.4 ± 8.0 (72.0)	76.8 ± 7.4 (77.0)	78.8 ± 8.1 (79.0)	76.2 ± 7.8 (76.5)	79.3 ± 8.1 (80.0)
Race, n (%)						
White	424 (72.0)	0 (0.0)	424 (100.0)	296 (72.9)	0 (0.0)	296 (98.3)
Black	98 (16.6)	98 (100.0)	0 (0.0)	74 (18.2)	74 (100.0)	0 (0.0)
Other	0 (0.0)	0 (0.0)	0 (0.0)	5 (1.2)	0 (0.0)	5 (1.7)
Unknown	67 (11.4)	0 (0.0)	0 (0.0)	31 (7.6)	0 (0.0)	0 (0.0)
Year of index date, n (%)						
2018	0 (0.0)	0 (0.0)	0 (0.0)	161 (39.7)	20 (27.0)	123 (40.9)
2019	51 (8.7)	7 (7.1)	33 (7.8)	113 (27.8)	20 (27.0)	87 (28.9)
2020	186 (31.6)	30 (30.6)	139 (32.8)	60 (14.8)	16 (21.6)	41 (13.6)
2021	288 (48.9)	42 (42.9)	213 (50.2)	54 (13.3)	15 (20.3)	36 (12.0)
2022	64 (10.9)	19 (19.4)	39 (9.2)	18 (4.4)	3 (4.1)	14 (4.7)
Time between castration resistance and index date, mo, median (IQR) (nmCRPC only)	–	–	–	8.7 (1.9, 25.7)	15.6 (4.9, 35.2)	7.8 (1.0, 21.9)
Time between metastasis and index date, mo, median (IQR) (mCSPC only)	1.5 (0.5, 4.9)	2.2 (0.9, 5.7)	1.5 (0.5, 5.0)	–	–	–
Prior use of ADT,^a^ n (%)	533 (90.5)	92 (93.9)	385 (90.8)	390 (96.1)	72 (97.3)	290 (96.3)
Time between ADT initiation and index date, mo, median (IQR)	3.2 (1.1, 22.3)	3.4 (1.1, 14.6)	3.3 (1.2, 26.4)	48.7 (26.7, 82.9)	47.9 (30.6, 91.9)	47.0 (26.5, 81.8)
Concurrent use of ADT,^b^ n (%)	567 (96.3)	94 (95.9)	407 (96.0)	374 (92.1)	69 (93.2)	279 (92.7)
Prior use of first-generation anti-androgens,^c^ n (%)	67 (11.4)	9 (9.2)	49 (11.6)	57 (14.0)	13 (17.6)	42 (14.0)
Prior use of bone antiresorptive therapy,^d^ n (%)	150 (25.5)	20 (20.4)	119 (28.1)	122 (30.0)	14 (18.9)	103 (34.2)
PSA test within 13 wk prior to and including the index date, n (%)	478 (81.2)	75 (85.2)	351 (90.5)	367 (90.4)	65 (90.3)	272 (93.5)
PSA level (ng/mL), mean ± SD (median)	19.2 ± 43.3 (3.4)	22.7 ± 50.3 (3.3)	18.3 ± 42.1 (3.1)	9.0 ± 16.1 (3.6)	13.7 ± 23.4 (5.5)	7.6 ± 13.5 (3.3)
Gleason score,^e^ n (%)						
≤6	28 (4.8)	4 (4.1)	21 (5.0)	30 (7.4)	8 (10.8)	21 (7.0)
7	103 (17.5)	15 (15.3)	76 (17.9)	68 (16.7)	12 (16.2)	49 (16.3)
8	68 (11.5)	10 (10.2)	51 (12.0)	33 (8.1)	9 (12.2)	22 (7.3)
9	130 (22.1)	20 (20.4)	91 (21.5)	55 (13.5)	10 (13.5)	42 (14.0)
10	9 (1.5)	0 (0.0)	9 (2.1)	10 (2.5)	0 (0.0)	7 (2.3)
Not available	251 (42.6)	49 (50.0)	176 (41.5)	210 (51.7)	35 (47.3)	160 (53.2)

^a^Prior use of ADT medication was defined as any ADT administration at any time prior to (and excluding) the index date.

^b^Concurrent use of ADT medication was defined as any ADT administration observed 180 days before or after the index date.

^c^Prior use of first-generation antiandrogens was defined as any prescription for bicalutamide, nilutamide, or flutamide, at any time prior to (and excluding) the index date.

^d^Prior use of bone antiresorptive therapy was defined at any time prior to (and excluding) the index date

^e^Gleason score was evaluated at any time prior to and including the index date, with the most recent value reported.

Abbreviations: ADT, androgen deprivation therapy; IQR, interquartile range; mCSPC, metastatic castration-sensitive prostate cancer; nmCRPC, non-metastatic castration-resistance prostate cancer; PSA, prostate-specific antigen; SD, standard deviation.

Concurrent ADT use with apalutamide was observed in most patients with mCSPC (overall: 96.3%; Black, 95.9%; non-Black, 96.0%) and nmCRPC (overall: 92.1%; Black: 93.2%; non-Black: 92.7%). Few patients had used first-generation anti-androgens (mCSPC: overall: 11.4%; Black: 9.2% non-Black: 11.6%; nmCRPC: overall, 14.0%; Black, 17.6%; non-Black, 14.0%) or bone antiresorptive therapy (mCSPC, overall: 25.5%; Black, 20.4%; non-Black, 28.1%; nmCRPC, overall: 30.0%; Black, 18.9%; non-Black, 34.2%).

### Persistence and Adherence

**Figure 1.**
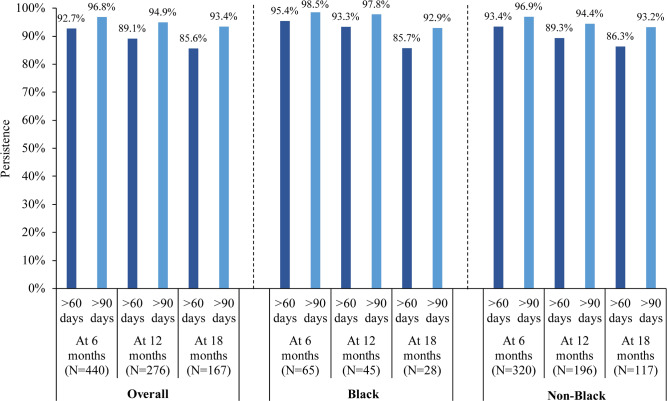
Apalutamide Persistence Using Treatment Gaps of >60 and >90 Days of Coverage Among Patients With mCSPC on Apalutamide

**Figure 2.**
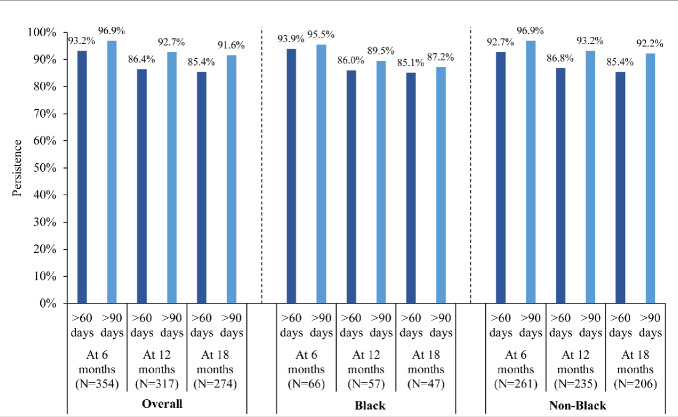
Apalutamide Persistence Using Treatment Gaps of >60 Days and >90 Days of Coverage among Patients With nmCRPC on Apalutamide

The median follow-up duration was 11.2 months in the mCSPC cohort and 27.0 months in the nmCRPC cohort. In the mCSPC cohort, treatment persistence at 12 months using a treatment gap of over 60 days and over 90 days was 89.1% and 94.9% overall, 93.3% and 97.8% for Black patients, and 89.3% and 94.4% for non-Black patients (**Figure 1**). In the nmCRPC cohort, treatment persistence at 12 months using a treatment gap of over 60 days and over 90 days was 86.4% and 92.7% overall, 86.0% and 89.5% for Black patients, and 86.8% and 93.2% for non-Black patients (**Figure 2**). Treatment persistence with apalutamide at other time points (eg, 6 and 18 months) are reported in **Figures 1 and 2**. Among patients with mCSPC, 65.5% were adherent at 6 months (Black, 64.6%; non-Black, 66.9%), 54.0% at 12 months (Black, 48.9%; non-Black, 56.1%) and 47.9% at 18 months (Black, 53.6%; non-Black, 47.0%). Among patients with nmCRPC, 59.3% were adherent at 6 months (Black, 59.1%; non-Black, 59.8%), 50.8% at 12 months (Black, 59.6%; non-Black, 50.6%) and 45.6% at 18 months (Black, 51.1%; non-Black, 46.6%).

### Clinical Outcomes

**Figure 3.**
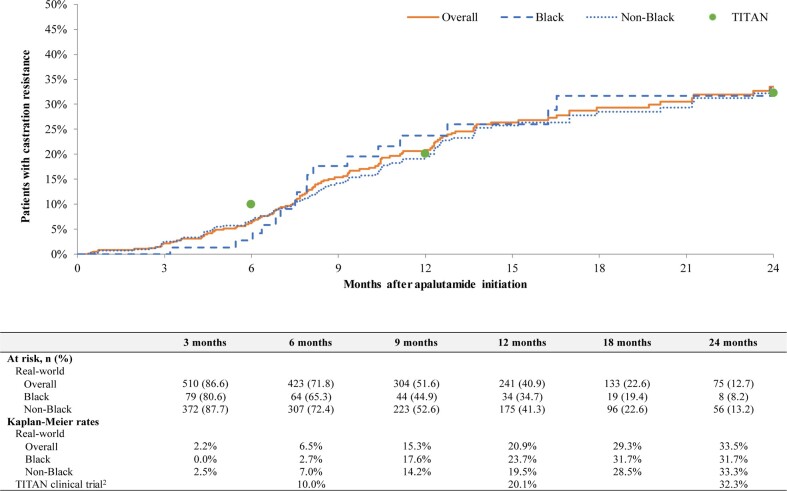
Progression to Castration Resistance Among Patients With mCSPC on Apalutamide^1^ ^1^Patients who did not develop castration resistance were censored at the earliest of end of clinical activity or data availability (1 April 2022). ^2^TITAN trial progression to castration resistance rates among the intent-to-treat population^18^ (Janssen Scientific Affairs, LLC, a Johnson & Johnson company data on file).

**Figure 4.**
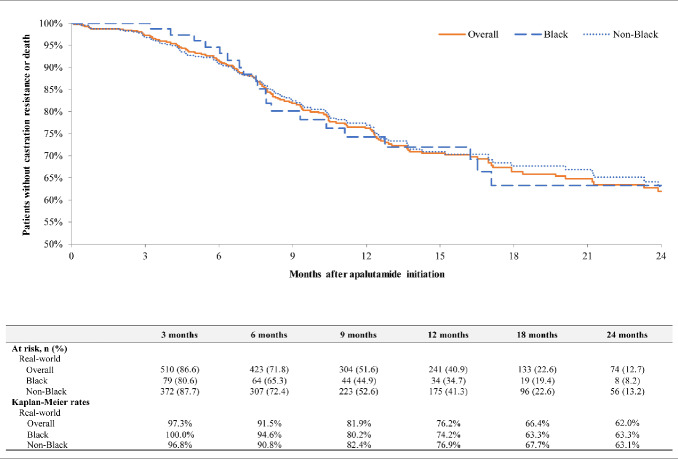
Castration Resistance-free Survival Among Patients with mCSPC on Apalutamide^1^ ^1^Time to castration resistance or death was assessed from the index date to the earliest of castration resistance or death. Patients without the event were censored at the earliest of end of clinical activity or data availability (1 April 2022).

**Figure 5.**
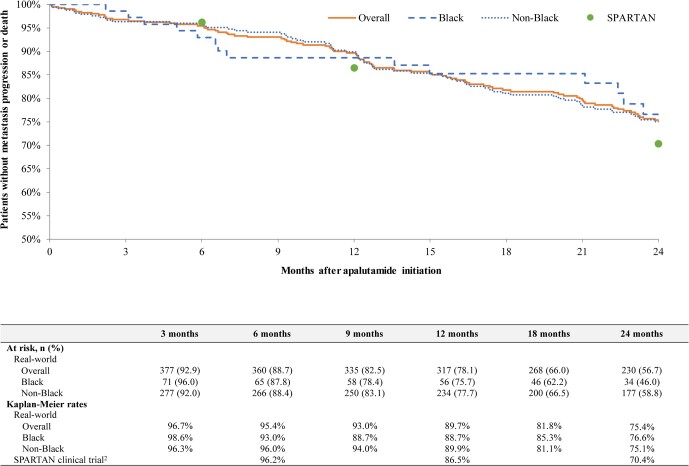
Metastasis-free Survival Among Patients with nmCRPC on Apalutamide^1^ ^1^Metastasis-free survival was assessed from the index date to the earliest of metastasis (ie, date of a confirmed bone, nodel, or visceral metastasis) or death. Patients without the event were censored at the earliest of end of clinical activity or data availability (1 April 2022). ^2^SPARTAN trial metastasis-free survival rates among the intent-to-treat population^11^ (Janssen Scientific Affairs, LLC, a Johnson & Johnson company data on file).

Among patients with mCSPC, overall progression to CR rates were 6.5% at 6 months (Black, 2.7%; non-Black, 7.0%), 20.9% at 12 months (Black, 23.7%; non-Black, 19.5%), and 33.5% at 24 months (Black, 31.7%; non-Black, 33.3%) (**Figure 3**). In the same cohort, overall CRFS rates were 91.5% at 6 months (Black, 94.6%; non-Black, 90.8%), 76.2% at 12 months (Black, 74.2%; non-Black, 76.9%), and 62.0% at 24 months (Black, 63.3%; non-Black, 63.1%) (**Figure 4**). In patients with nmCRPC, overall MFS rates were 95.4% at 6 months (Black, 93.0%; non-Black, 96.0%), 89.7% at 12 months (Black, 88.7%; non-Black, 89.9%), and 75.4% at 24 months (Black, 76.6%; non-Black, 75.1%) (**Figure 5**). Median time to MFS, progression to CR, and CRFS were not reached in any group.

## DISCUSSION

While clinical trials have demonstrated the efficacy and safety of apalutamide,^11,12^ there is limited real-world data describing treatment patterns and clinical outcomes among patients with mCSPC and nmCRPC who initiated apalutamide. In this study, the majority of the population with mCSPC and nmCRPC demonstrated persistence up to 18 months of observation, indicating low levels of discontinuation of apalutamide. Furthermore, this study provides information about treatment patterns and clinical outcomes in Black patients, a population that experiences a greater burden of advanced PC and often has poor representation in clinical trials.^8,11,12,15,16^ In this study, treatment patterns and clinical outcomes, including progression to CR and MFS, were descriptively similar among Black and non-Black men, and the clinical outcomes were consistent with those observed in the TITAN and SPARTAN trials.^11,12,20^

Treatment persistence can be an important indicator of the effectiveness and tolerability of a medication. In this study, treatment persistence at 12 months of follow-up remained in over 85% of the mCSPC and nmCRPC cohorts and was descriptively similar between Black and non-Black patients. This finding is notable considering previous evidence has demonstrated that Black race is an important factor influencing advanced PC medication adherence and persistence.^21^ In another real-world study by Lowentritt et al,^15^ a high proportion of patients with nmCRPC also demonstrated treatment persistence, albeit slightly lower levels than in the current study; for example, using a treatment gap of over 90 days, Lowentritt et al reported 6- and 12-month persistence rates of 88.9% and 69.6%, respectively,^15^ compared with 96.9% and 94.9% in this current study. Maintaining high levels of treatment persistence is an important treatment goal that may ultimately help improve clinical outcomes.

In the present study, rates of progression to CR among patients with mCSPC and the MFS rates among patients with nmCRPC were consistent with results observed in the TITAN and SPARTAN phase 3 clinical trials, respectively.^11,20^ In the TITAN trial, evaluating the efficacy of apalutamide in patients with mCSPC, the 24-month progression to CR rate was 32.3% (Janssen Scientific Affairs, LLC, a Johnson & Johnson company, data on file),^20^ compared with 33.5% in this study. Additionally, the median time to CR was not reached in either the TITAN trial^12,20^ or this study, and CRFS is not reported in the TITAN trial. To our knowledge, CR progression rates and CRFS have only been reported in one other real-world study of patients with mCSPC initiating apalutamide.^22^ Using EMR data from the Flatiron oncology network, Lowentritt et al observed that 27.9% of patients with mCSPC progressed to CR by 24 months and 65.9% did not progress to CR or die by 24 months, which was similar to those observed in this current study.^22^ In the SPARTAN trial, a study that evaluated the efficacy of apalutamide in patients with nmCRPC, the MFS rate at 24 months was 70.4% (Janssen Scientific Affairs, LLC, a Johnson & Johnson company data on file),^11^ compared with 75.4% in this study. In addition, median time to MFS was 40.5 months in the SPARTAN trial and was not reached by a median follow-up duration of 27.0 months in this study.^11^ To our knowledge, no other published real-world studies have reported rates of MFS among patients with nmCRPC.

Disparities among Black men with PC regarding representation in clinical trials, access to treatment, and clinical outcomes have been observed.^1,8,23^ An analysis of PC clinical trial enrollment reported that roughly less than 5% of trial participants are Black, though they represent almost 13% of the US population.^8^ In line with these observations, Black men represented a small proportion of patients in the TITAN trial (1.8% Black patients) and the SPARTAN trial (5.6% Black patients),^13,14^ limiting the ability to make inferences in this racial subgroup from the respective trials. In this study, 17.3% of men were Black, which is consistent with the demographics reported in Lowentritt et al (17.1% Black),^15^ and overall, more aligned with the US real-world population.^24^

A notable finding in this study is the observation of a longer delay between metastasis and apalutamide initiation (Black, 2.2 months; non-Black, 1.5 months), as well as between CR and apalutamide initiation (Black, 15.6 months; non-Black: 7.8 months), for Black patients, highlighting a potential disparity between diagnosis and treatment onset. Delays between diagnosis and treatment initiation in Black patients with PC have previously been reported, including a retrospective analysis by Kinlock et al that revealed Black men with PC were more likely to experience longer delays between diagnosis and treatment than White men.^25^ Similarly, Cobran et al reported that Black men had a longer median time to ADT treatment after diagnosis of metastatic PC than White men.^23^ However, despite the longer delay between diagnosis and apalutamide initiation in Black men observed in this study, clinical outcomes, notably, progression to CR, CRFS, and MFS were similar among Black and non-Black patients. This finding provides some evidence that apalutamide is still effective, even among patients who experience a delay in treatment initiation.

Comparable treatment outcomes among Black and non-Black patients have been observed in previous studies.^26,27^ While not evaluated in the current study, OS and PFS were comparable between Black and non-Black patients in a real-world study of patients with mCSPC receiving either docetaxel or abiraterone acetate plus prednisone.^27^ Taken together, these study findings fill an important knowledge gap, reporting race-specific data demonstrating similar real-world effectiveness of apalutamide among Black and non-Black patients.

### Limitations

The results of this study are subject to certain limitations. Inaccuracies or omissions (eg, diagnosis dates, treatment start dates) are possible with the clinical data used in this study. In addition, data were limited to diagnoses, medical services, or prescription fills acquired within the selected network of community-based urology practices, while diagnoses, services and prescriptions obtained outside the network were not captured. Moreover, the generalizability of the study may be limited because the sample was restricted to patients who received medications through in-office dispensations in community urology practices and, therefore, may not be representative of the entire population of patients with mCSPC or nmCRPC in the US.

## CONCLUSION

In this real-world study of patients with mCSPC or nmCRPC treated in a community-based urology setting, apalutamide demonstrated robust real-world effectiveness consistent with the TITAN trial (with respect to progression to CR) and the SPARTAN trial (with respect to MFS), and treatment persistence and adherence remained high throughout follow-up. Furthermore, this study also demonstrated that clinical outcomes and treatment patterns in Black men with mCSPC or nmCRPC are descriptively similar to those obtained in non-Black men.

## Supplementary Material

Online Supplementary Material
