# Supplementary material for: Real-World Clinical Outcomes and Treatment Patterns Among Black and Non-Black Patients With Prostate Cancer Initiated on Apalutamide in a Urology Setting
Source: J Health Econ Outcomes Res. 2024 Aug 19;11(2):41–8. doi: 10.36469/001c.121233 (PMC11392484; doi:10.36469/001c.121233)
Supplement: Online Supplementary Material [file jheor_2024_11_2_121233_242274.pdf]

Journal of Health Economics and Outcomes Research

Online Supplementary Material

Real-World Clinical Outcomes and Treatment Patterns Among Black and Non-Black Patients With Prostate Cancer Initiated on Apalutamide in a Urology Setting. *JHEOR*. 2024;11(2):41-48. doi:10.36469/jheor.2024.121233

Figure S1: Patient Flowchart

This supplementary material has been provided by the authors to give readers additional information about their work.

This is an open-access article distributed under the terms of the Creative Commons Attribution 4.0 International License (CCBY-4.0). View this license's legal deed at http://creativecommons.org/licenses/by/4.0 and legal code at http://creativecommons.org/licenses/by/4.0/legalcode for more information.

Supplementary Figure 1. Patient Flowchart

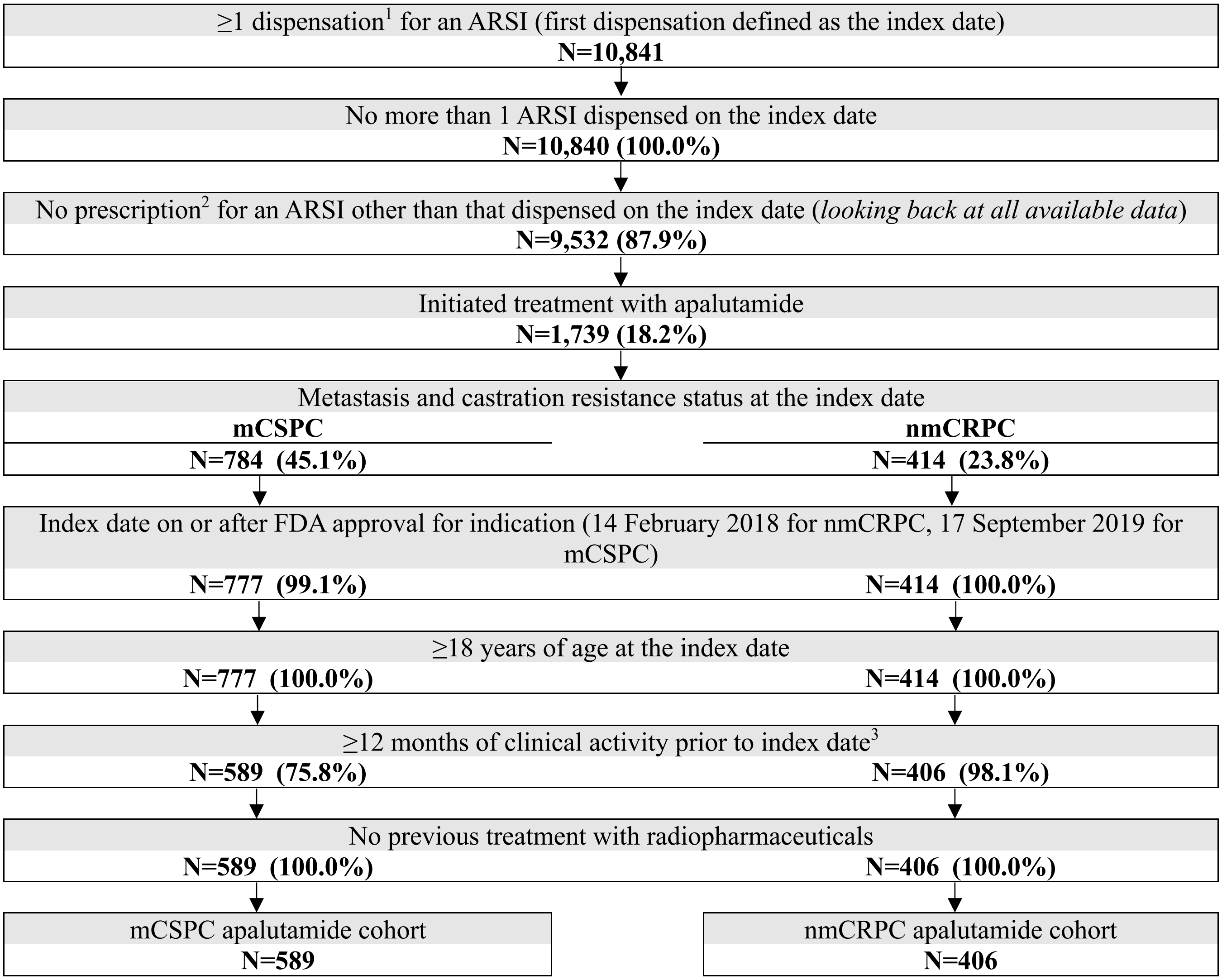

ARSI: androgen receptor signaling inhibitor; FDA: Food and Drug Administration; mCSPC: metastatic castration-sensitive prostate cancer; nmCRPC: non-metastatic castration-resistant prostate cancer

Notes:

1. Agent identified at index date was based on medication dispensations only.
2. To confirm that patients were newly initiated on an ARSI as identified through dispensations, prescription information was examined to exclude patients with prescriptions for ARSIs other than apalutamide prior to the index date.
3. Continuous clinical activity was defined as the period from the first to last record in the clinical database. Patients with no observation period after the index date were excluded.
